# Clostridium butyricum and Its Derived Extracellular Vesicles Modulate Gut Homeostasis and Ameliorate Acute Experimental Colitis

**DOI:** 10.1128/spectrum.01368-22

**Published:** 2022-06-28

**Authors:** Lingyan Ma, Qicheng Shen, Wentao Lyu, Lu lv, Wen Wang, Minjie Yu, Hua Yang, Shiyu Tao, Yingping Xiao

**Affiliations:** a State Key Laboratory for Managing Biotic and Chemical Threats to the Quality and Safety of Agro-products, Institute of Agro-product Safety and Nutrition, Zhejiang Academy of Agricultural Sciences, Hangzhou, China; b College of Biotechnology and Bioengineering, Zhejiang University of Technology, Hangzhou, China; c College of Animal Sciences and Technology, Huazhong Agricultural University, Wuhan, China; Lerner Research Institute

**Keywords:** colitis, gut microbiota, gut barrier function, *Clostridium butyricum*, extracellular vesicles

## Abstract

Microbiological treatments are expected to have a role in the future management of inflammatory bowel disease (IBD). Clostridium butyricum (*C. butyricum*) is a probiotic microorganism that exhibits beneficial effects on various disease conditions. Although many studies have revealed that C. butyricum provides protective effects in mice with colitis, the way C. butyricum establishes beneficial results in the host remains unclear. In this study, we investigated the mechanisms by which C. butyricum modifies the gut microbiota, produces bacterial metabolites that may be involved, and, specifically, how microbial extracellular vesicles (EVs) positively influence IBD, using a dextran sulfate sodium (DSS)-induced colitis murine model in mice. First, we showed that C. butyricum provides a protective effect against colitis, as evidenced by the prevention of body weight loss, a reduction in the disease activity index (DAI) score, a shortened colon length, decreased histology score, and an improved gut barrier function, accompanied by reduced levels of pathogenic bacteria, including Escherichia/Shigella, and an increased relative abundance of butyrate-producing Clostridium sensu stricto-1 and *Butyricicoccus*. Second, we also confirmed that the gut microbiota and metabolites produced by C. butyricum played key roles in the attenuation of DSS-induced experimental colitis, as supported by the profound alleviation of colitis effects following fecal transplantation or fecal filtrate insertion supplied from C. butyricum-treated mice. Finally, C. butyricum-derived EVs protected the gut barrier function, improved gut microbiota homeostasis in ulcerative colitis, and contributed to overall colitis alleviation.

**IMPORTANCE** This study indicated that C. butyricum provided a prevention effect against colitis mice, which involved protection of the intestinal barrier and positively regulating gut microbiota. Furthermore, we confirmed that the gut microbiota and metabolites that were induced by C. butyricum also contributed to the attenuation of DSS-induced colitis. Importantly, C. butyricum-derived EVs showed an effective impact in alleviating colitis.

## INTRODUCTION

The incidence of inflammatory bowel disease (IBD) is rising worldwide, represented by ulcerative colitis (UC) and Crohn’s disease (CD), which coincides with a marked change in environmental factors (1, 2). A consensus of evidence reveals that IBD is associated with dysbiosis of the gut microflora (3, 4). Dysbiosis alters not only the intestinal microbiota community composition but also its metabolome, exerting a wide range of negative influences on host cells (5). Thus, microbial-based treatments may have a key role in the management of IBD involving treatments centered around the use of fecal microbiota transplantation (FMT) and probiotics (6–9).

Intestinal epithelial barrier disruption is a characteristic feature of IBD, which leads to increased permeability and infiltration of pathogens (10). The mucus layer is thinner and less continuous in UC patients. Such aberrations in the protective mucus barrier increase a host’s susceptibility to developing colitis (11). Epithelial tight junctions (TJs) are constructed with both transmembrane proteins and peripheral membrane proteins, such as occludin, different claudins, and the zona occludens (ZO)-1, ZO-2, and ZO-3 (12). Patients with IBD can experience diminished levels of one or more of these proteins, resulting in impaired TJ functions and membrane dysfunction (13). These barrier defects compromise the immune system, subsequently causing the development of IBD.

Studies have demonstrated that probiotics help to modulate intestinal inflammation, which in turn may alleviate disease (14). A specific strain of the Gram-positive, butyric acid-producing bacterium, Clostridium butyricum, is a probiotic known to help mitigate symptoms of IBD. It possesses immunomodulatory and anti-inflammatory characteristics (15). C. butyricum is a fermentative bacterium that can degrade undigested dietary fiber, generating short-chain fatty acids (SCFAs), specifically butyrate (16). As such, C. butyricum has become an attractive candidate for the provision of beneficial effects on a host’s gut homeostasis. Although many publications have revealed the protective effects of C. butyricum in mice with colitis, understanding the interactions of gut microorganisms and their metabolites with C. butyricum requires further investigation. Moreover, determining how C. butyricum communicates with host cells and exerts influence over the gut microbial community needs to be investigated.

Probiotic-derived extracellular vesicles (EVs) may also serve as modulators of host immunity and intestinal barrier function (17). EVs appear to deliver microbial molecules to distant target cells in the host. They contain multimolecular complexes that have a regulatory influence on intercellular signaling, thus performing a critical role in mediating bacterium-host communication (17, 18). EVs released from pathogens can deliver immunostimulatory substances and toxins to host cells, which contribute to bacterial pathogenesis (19). Conversely, EVs from probiotics and other beneficial bacteria may enhance host immune status, modulate intestinal barrier strength, and provide other favorable effects (20). Because the function of C. butyricum-derived EVs has not yet been fully characterized, we further explored whether EVs from C. butyricum are likely to contribute to C. butyricum*-*mediated protective effects against the progression of UC.

In this study, we revealed the preventative effects of C. butyricum supplementation on intestinal barrier protection and the gut microbiota regulation in a murine model of dextran sulfate sodium (DSS)-induced colitis in mice. FMT and sterile fecal filtrate (FFT) experiments confirmed the potential beneficial effects of the C. butyricum-mediated microbiota community and its metabolites in mice with colitis. Finally, we showed that C. butyricum-derived EVs inhibited the proinflammatory response, alleviating intestinal barrier damage and ultimately ameliorating acute colitis.

## RESULTS

### C. butyricum pretreatment attenuated DSS-induced colitis.

**FIG 1:**
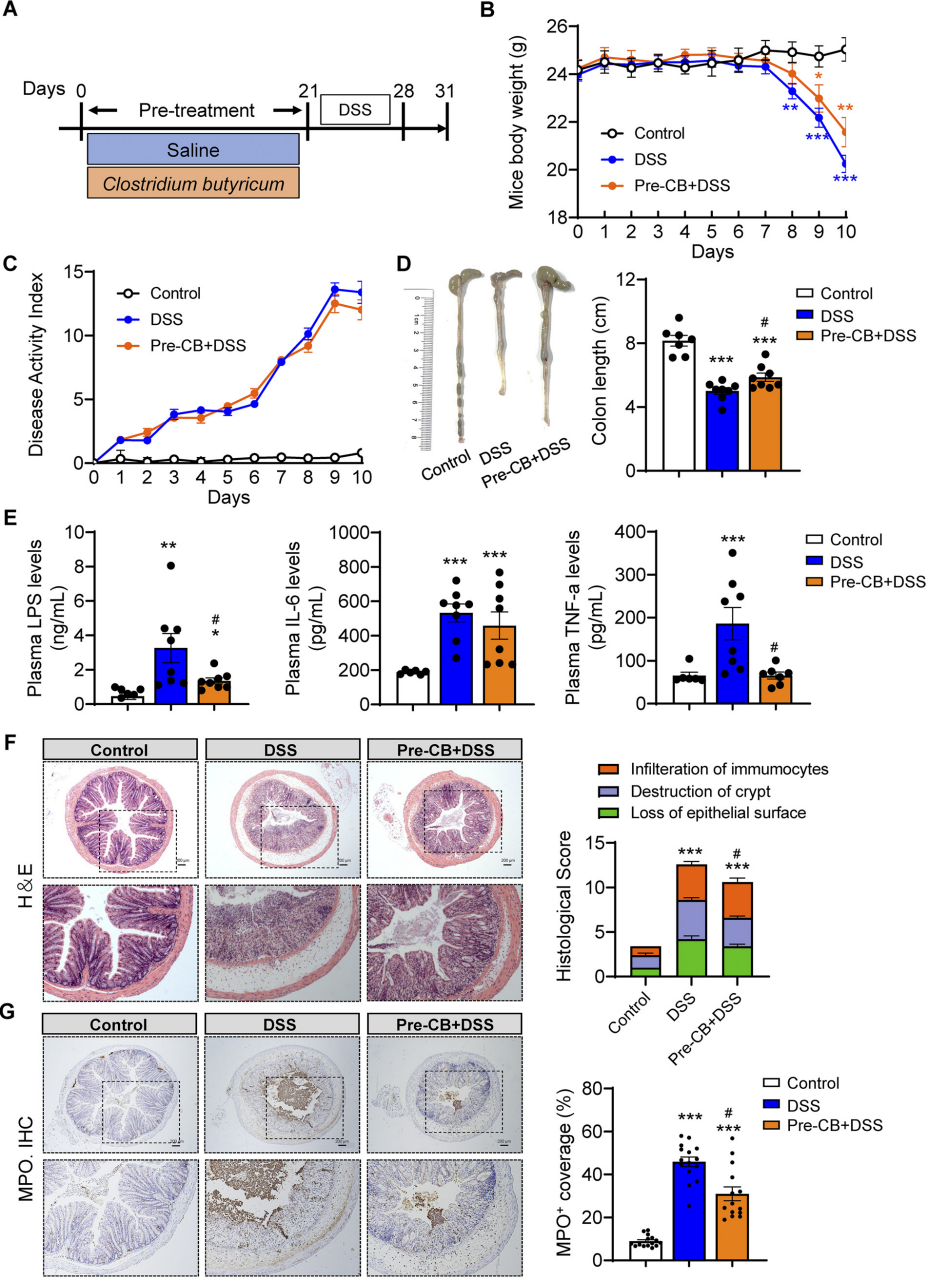
C. butyricum pretreatment attenuated colitis. (A) Experimental design. (B) Mice body weight (*n *= 8). (C) DAI score (*n *= 8). (D) Representative picture of the colon and colon length (*n *= 8). (E) Plasma LPS, IL-6, and TNF-α levels (*n *= 8). (F) H&E staining of the colon and summarized histological score (*n *= 5). (G) Immunohistochemical analysis of colonic inflammation marker MPO in the colonic section from mice and summarized MPO stained rate. Scale bar: 200 μm. *, *P ≤ *0.05; **, *P ≤ *0.01 versus the control group, ^#^, *P ≤ *0.05; ^##^, *P ≤ *0.01 versus the DSS group.

Mice were pretreated with C. butyricum for 21 days to determine the prevention impact of C. butyricum against colitis (Fig. 1A). Moreover, DSS mice experienced obvious weight loss (Fig. 1B). Of note, C. butyricum pretreatment slightly increased the body weight from day 7 to 10 compared with that of the colitis mice (Fig. 1B). The DAI score was slightly reduced in CB-pretreated mice compared with the DSS group (Fig. 1C). Furthermore, the average colon length shortening of the DSS group was remarkably reversed in CB-pretreated mice (Fig. 1D). C. butyricum significantly suppressed spleen enlargement in DSS-treated mice, which was associated with splenic macrophage infiltration (Fig. S1A).

On the other hand, plasma proinflammatory markers, including lipopolysaccharide (LPS), interleukin (IL)-6, and tumor necrosis factor (TNF)-α, were significantly increased in DSS-treated mice, while CB pretreatment decreased LPS and TNF-α levels (Fig. 1E). Moreover, the histological assessment revealed that CB pretreatment significantly reversed DSS-induced inflammatory infiltration, epithelial barrier damage, and crypt destruction (Fig. 1F). In addition, colonic myeloperoxidase (MPO) activity, a neutrophil-associated marker, was assessed using immunofluorescence microscopy. These results showed that, compared with the control group, DSS induced a higher level of MPO staining coverage, while CB pretreatment decreased the activity, as shown by a lower coverage rate (Fig. 1G). Considered together, these results suggest that pretreatment with C. butyricum could significantly ameliorate DSS-induced colitis phenotypes.

### C. butyricum pretreatment attenuated DSS-induced colonic barrier dysfunction.

**FIG 2:**
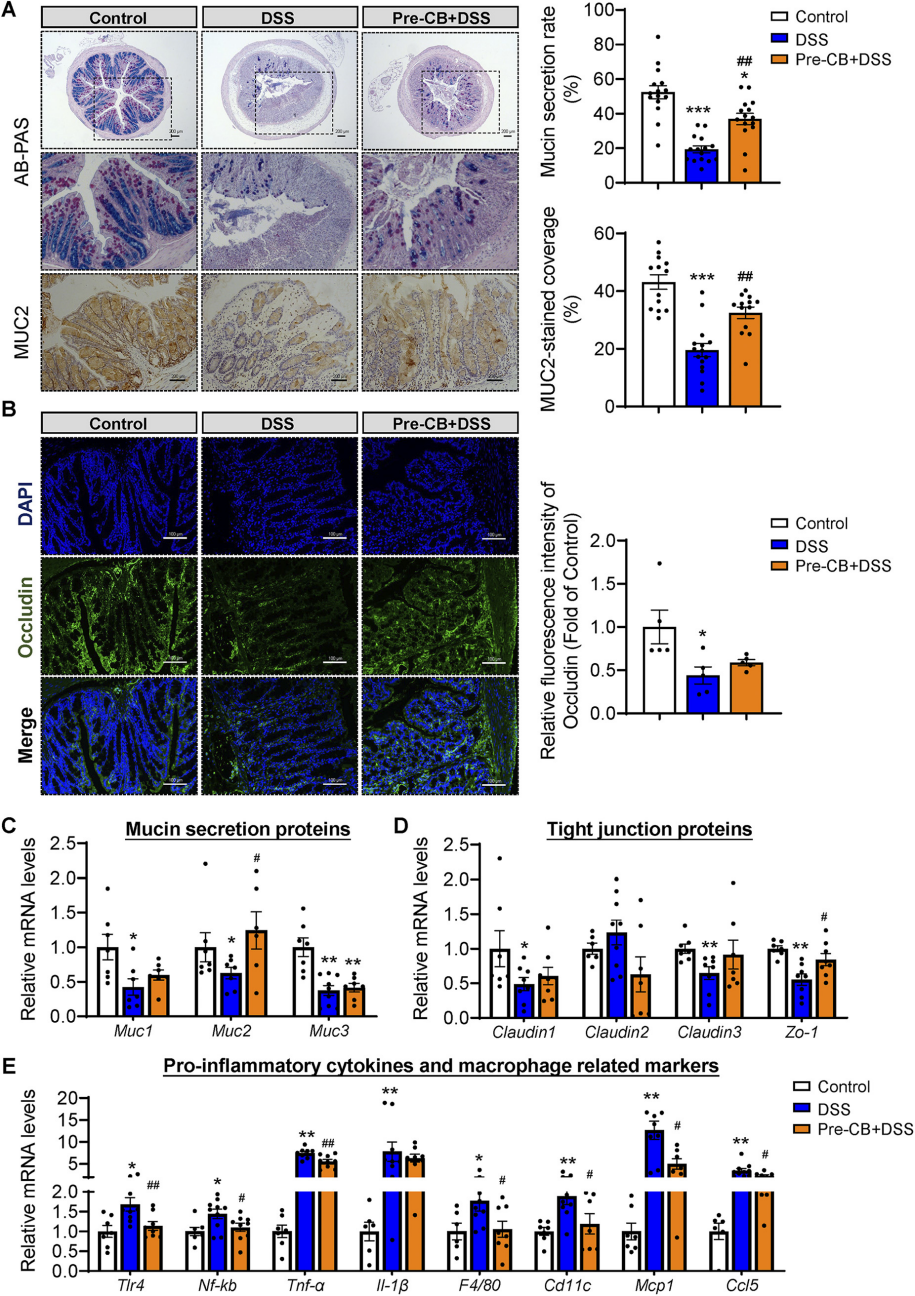
C. butyricum pretreatment attenuated the DSS-induced colonic barrier dysfunction. (A) Alican blue-PAS staining of the colon with its mucin secretion rate and immunohistochemical analysis of MUC2 with MUC2-stained coverage. Scale bar: 200 μm. (B) Immunofluorescence staining of tight junction protein Occludin expression in the colonic section from mice (*n *= 5). Blue: DAPI; Green: Occludin. (C to E) Relative mRNA levels of Mucin secretion proteins, tight junction proteins, and proinflammatory related gene expression (*n *= 8). *, *P ≤ *0.05; **, *P ≤ *0.01 versus the control group, ^#^, *P ≤ *0.05; ^##^, *P ≤ *0.01 versus the DSS group.

To further illuminate how C. butyricum pretreatment remitted colitis severity, colonic barrier function was assessed. Representative pictures of AB-PAS and immunohistochemical staining of MUC2 in the colon of each group are shown in Fig. 2A and B. Compared with the control group, crypt loss, mucosal surface damage, and a decrease in mucin protein secretion were found in the DSS group (Fig. 2A). However, C. butyricum pretreatment significantly improved the MUC2 protein secretion rate (Fig. 2A). Occludin, as a functional component of tight junction proteins, was upregulated by C. butyricum pretreatment, as detected by immunofluorescence (Fig. 2B).

We next quantified the expression of mucin secretion proteins (*Muc1*, *Muc2*, *Muc3*), tight junction proteins (*Claudin1*, *Claudin2*, *Claudin3*, *Zo-1*), and proinflammatory-related markers (*Tlr4*, *Nf-kb*, *Tnf-α*, *Il-1β*, *F4/80*, *Cd11c*, *Mcp1*, *Ccl5*) in the colonic samples by RT-PCR (Fig. 2C to E). In line with the above results, DSS administration resulted in a decrease in mucin proteins and tight junction protein expression and promoted proinflammatory signaling expression (Fig. 2C to E). More importantly, C. butyricum pretreatment significantly upregulated the gene expression of *Muc2* and *Zo-1* while downregulating the mRNA levels of *Tlr4* and *Nf-kb* (Fig. 2C to E). Additionally, the high expression of the proinflammatory cytokine *Tnf-α*, proinflammatory macrophage markers *F4/80* and *Cd11c*, and chemokines *Mcp1* and *Ccl5* induced by DSS was significantly ameliorated by C. butyricum. These results suggested that C. butyricum pretreatment protected DSS-induced colonic barrier dysfunction via the enhancement of epithelial tight junction and mucosal barrier integrity and the inhibition of the proinflammatory response.

### C. butyricum pretreatment regulated the gut microbiota composition in DSS-induced colitis.

**FIG 3:**
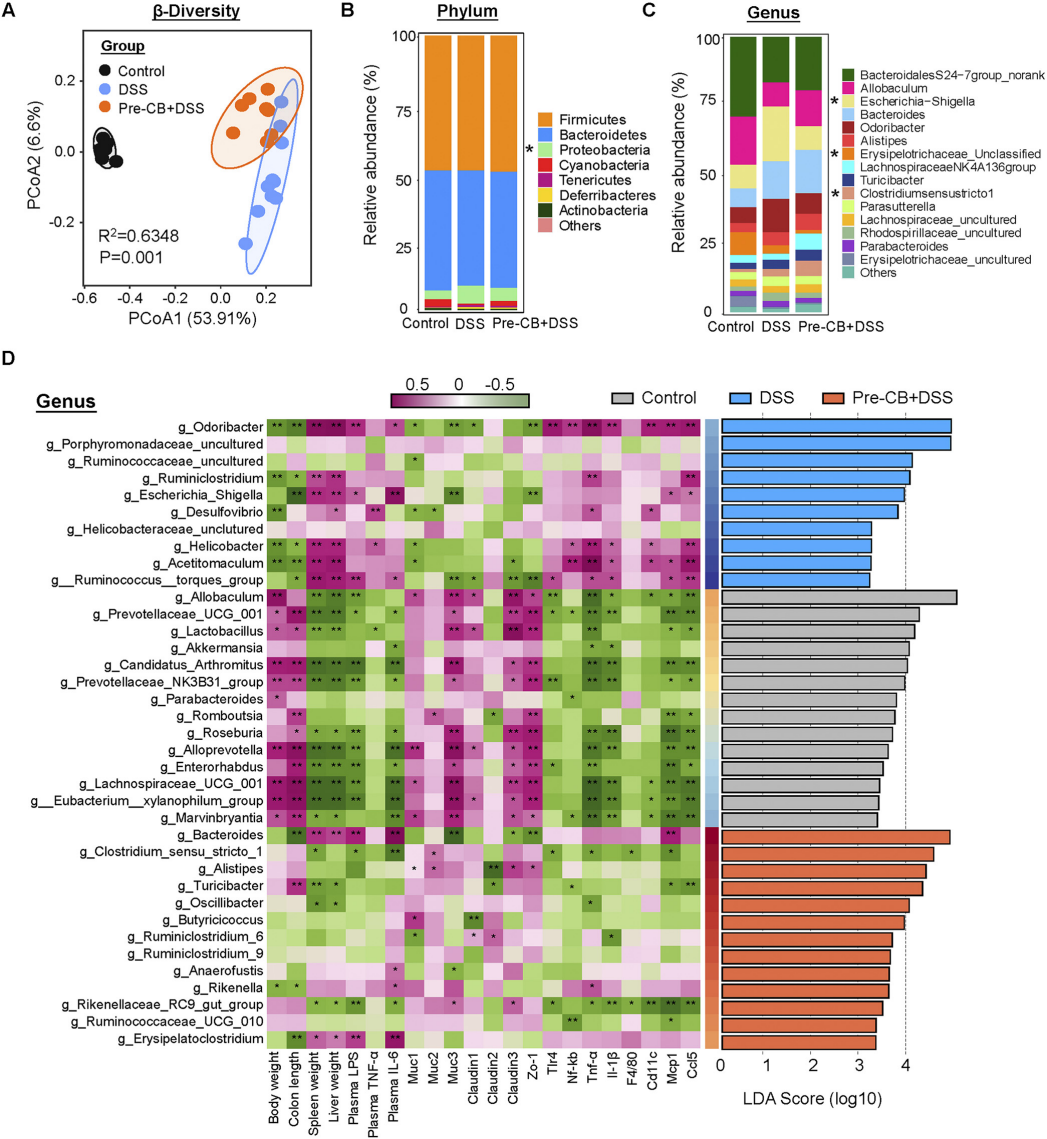
C. butyricum pretreatment regulated the gut microbiota composition in DSS-induced colitis. (A) PCoA of gut microbiota in each group (*n *= 8). (B) Phylum levels. (C) Genus levels. (D) Left: heatmap of Spearman correlation between intestinal microbiota and colitis-related phenotype traits in each group. Right: analysis of differences in the microbial taxa shown by LEfSe.

The colonic microbiota composition was evaluated to investigate whether C. butyricum could reverse DSS-induced dysbiosis. PCoA analysis showed separation between control mice and the colitis group, which was also observed between the Pre-CB+DSS and DSS groups (Fig. 3A). At the phylum level, the colonic microbiota was dominated by *Bacteroidetes*, *Firmicutes*, and *Proteobacteria.* Moreover, the abundance of *Proteobacteria* significantly increased in the DSS group, while C. butyricum pretreatment decreased in abundance (Fig. 3B). At the genus level, *Bacteroidales* S24-7group_norank, *Allobaculum*, Escherichia*/Shigella*, *Bacteroides*, and *Odoribacter* were the predominant genera (Fig. 3C). In addition, DSS induced a remarkable increase in the Escherichia*/Shigella* level, which was significantly reduced by C. butyricum treatment (Fig. 3C). Furthermore, Clostridium sense stricto-1 was increased by DSS and C. butyricum treatment (Fig. 3C). *Clostridium sense stricto* 1, *Alistipes*, and *Butyricicoccus* showed marked enrichment in the CB-administered groups by linear discriminant analysis effect size (LEfSe) (Fig. 3D). Harmful bacteria, such as *Odoribacter*, Escherichia*/Shigella*, and *Desulfovibrio*, were enriched in the gut microbiota of the DSS group, while the probiotics *Lactobacillus* and *Akkermansia* were more abundant in the control mice (Fig. 3D).

Spearman correlation analysis further confirmed that the gut microbiota was associated with colitis parameters. For example, *Odoribacter* and Escherichia*/Shigella*, which were enriched in DSS-induced mice, had a strong negative association with colon length and *Muc3* and *Zo-1* expression but a strong positive correlation with plasma LPS and IL-6 levels and colonic proinflammatory marker *Mcp1* and *Ccl5* expression (Fig. 3D). As expected, the probiotic *Lactobacillus* showed a remarkable positive connection with barrier function but was negatively related to proinflammatory cytokines and genes (Fig. 3D). Clostridium sensu stricto-1 and *Alistipes* were positively related to *Muc2* expression. However, *Butyricicoccus* was found to be negatively associated with colonic *Claudin1* expression (Fig. 3D). These results indicated that C. butyricum pretreatment beneficially regulated the gut microbiota composition after DSS-induced dysbiosis.

### C. butyricum-changed gut microbiota and bacterial metabolites contributed to alleviated colitis.

**FIG 4:**
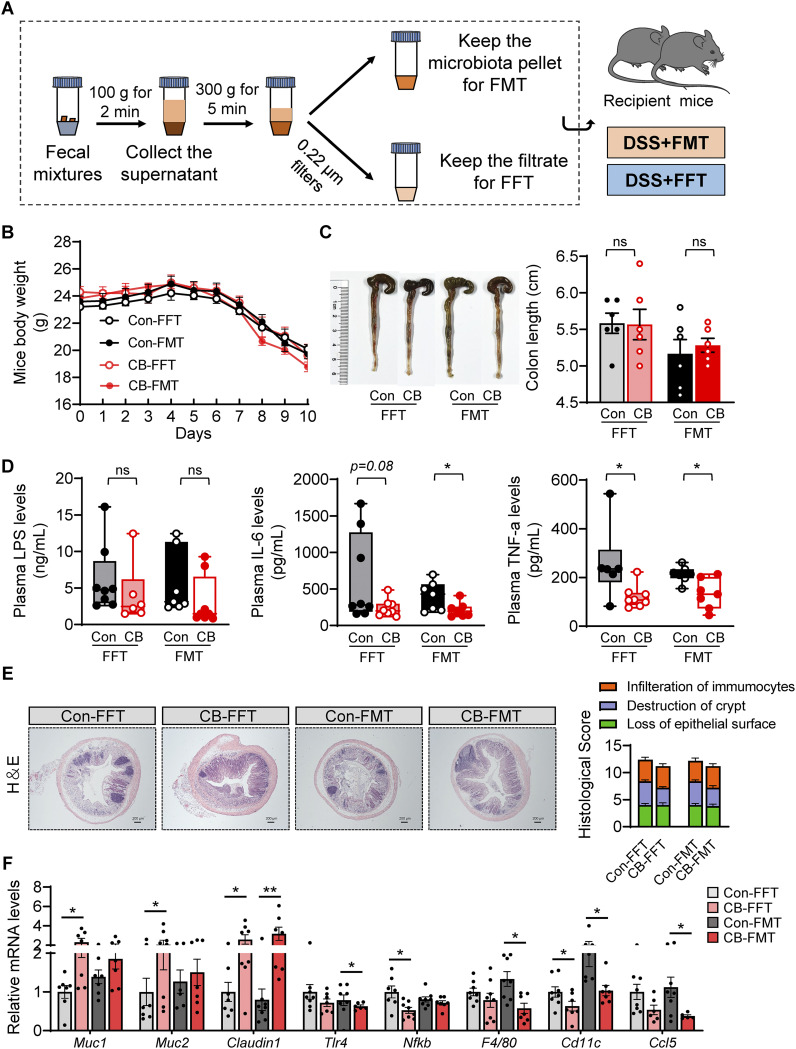
C. butyricum-changed gut microbiota and bacterial metabolites contributed to alleviated colitis. (A) Experimental design. (B) Mice body weight (*n *= 8). (C) Colon length. (D) Plasma LPS, IL-6, and TNF-α levels (*n *= 6 to 8). (E) H&E staining of the colon and summarized histological score (*n* = 5). (F) Relative mRNA levels of Mucin secretion proteins, tight junction proteins, and proinflammatory related gene expression (*n *= 6 to 8). *, *P ≤ *0.05; **, *P ≤ *0.01 versus the Con-FFT/Con-FMT group.

FMT and FFT were further performed to determine the involvement of gut microbiota and bacterial metabolites in CB-mediated colitis alleviation (Fig. 4A). The results suggested that FMT and FFT from CB-treated donor mice slightly minimized the body weight loss and colon length shortening compared to FMT or FFT from control-treated mice (Fig. 4B). In addition, FMT or FFT from CB-treated donor mice significantly or slightly reduced the spleen weight (Fig. S1B). However, the LPS levels showed no obvious differences between the groups (Fig. 4D). FMT from CB-treated donor mice might contribute more to decreasing plasma inflammation levels, as evidenced by significantly reduced IL-6 and TNF-α levels (Fig. 4D). Moreover, the histological assessment revealed that FMT or FFT from CB-treated donor mice ameliorated immunocyte infiltration, crypt destruction, and loss of epithelial surface (Fig. 4E). Furthermore, RT-PCR results suggested that FFT from CB-treated donor mice significantly upregulated the expression of *Muc1* and *Muc2* and downregulated the levels of *Nf-kb* and *Cd11c* compared with the FFT from control-treated mice (Fig. 4F). On the other hand, FMT from CB-treated donor mice notably increased the expression of *Claudin1* and reduced *Tlr4*, *F4/80*, and *Ccl5* expression (Fig. 4F). However, other genes, such as *Muc4*, *Claudin2*, *Claudin3*, *Zo-1*, *Mcp1*, *Tnf-α,* and *Il-1β*, showed no obvious difference between the two groups (Fig. S2). Collectively, C. butyricum-changed gut microbiota and metabolites were able to alleviate DSS-induced colitis.

### C. butyricum-changed gut microbiota and bacterial metabolites modulated gut microbiota in colitis mice.

**FIG 5:**
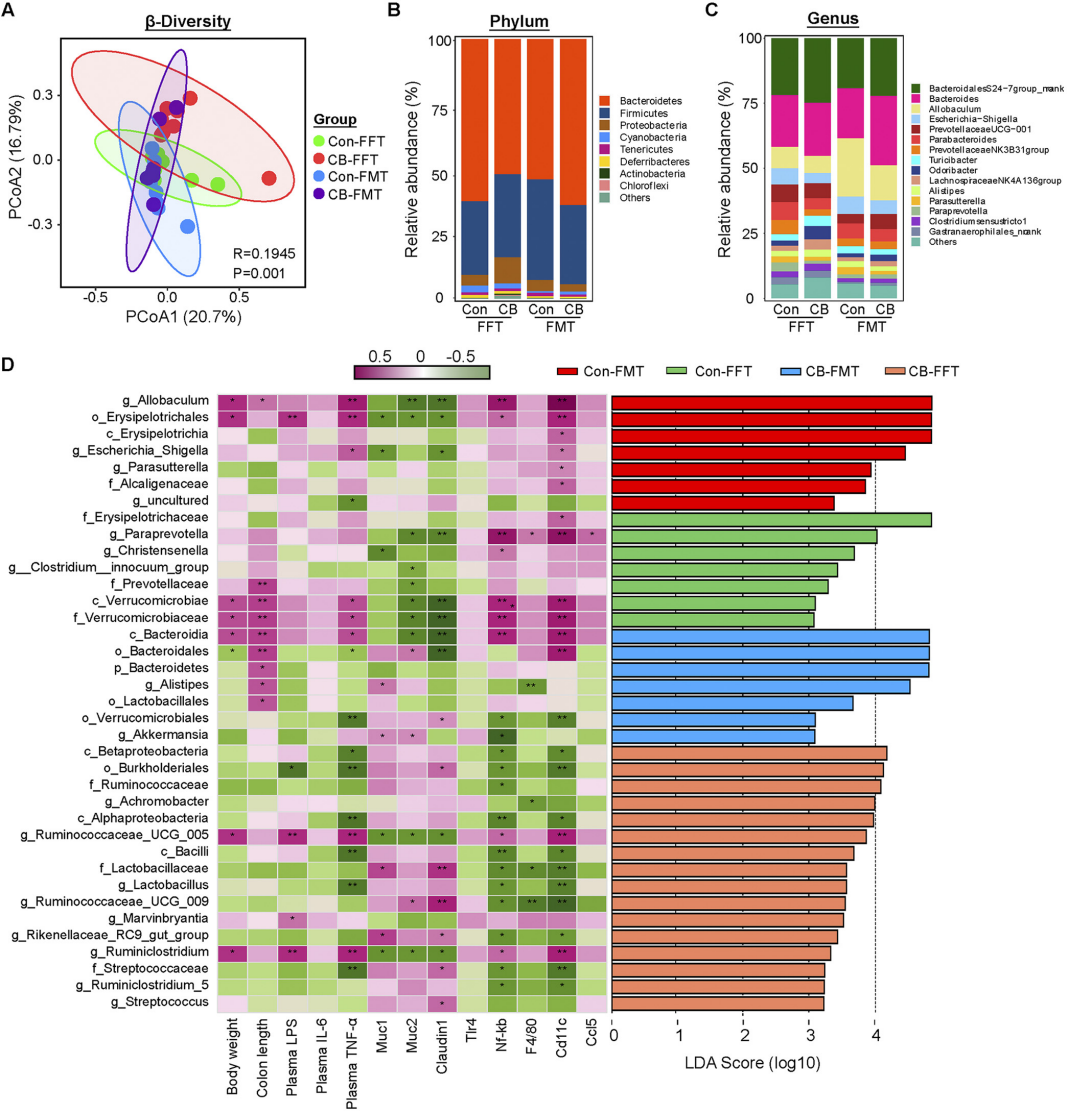
C. butyricum-changed gut microbiota and bacterial metabolites modulated gut microbiota in colitis mice. (A) PCoA of gut microbiota in each group (*n *= 8). (B) Phylum levels. (C) Genus levels. (D) Left: heatmap of Spearman correlation between intestinal microbiota and colitis-related phenotype traits in each group. Right: analysis of differences in the microbial taxa shown by LEfSe.

The gut microbiota changes resulting from FMT or FFT were further examined. The PCoA (principal coordinates analysis) results showed the change in microbiota structure among all the groups (Fig. 5A). The main composition did not change significantly among the groups at the phylum and genus levels (Fig. 5B and C). The phylum *Proteobacteria* was increased in the CB-FFT group but decreased in the CB-FMT group (Fig. 5B). Of note, Escherichia*/Shigella* were significantly reduced by CB-FFT and CB-FMT (Fig. 5C). Spearman correlation analysis and LEFSe results showed that the genus *Allobaculum* was enriched in the Con-FMT group. This positively correlated with plasma TNF-α levels and *Nf-kb* and *Cd11c* expression but negatively correlated with *Muc2* and *Claudin1* expression (Fig. 5D). In addition, the genus *Paraprevotella* was abundant in the Con-FFT group. It showed a negative association with *Muc2* and *Claudin1* expression and a positive relationship with proinflammatory genes (Fig. 5D). More importantly, *Akkermansia* was positively related to *Muc1* and *Muc2*, which were found in the CB-FMT group (Fig. 5D). The CB-FFT group was dominated by *Burkholderiales*, *Ruminococcaceae*, *Achromobacter*, and *Lactobacillus.* These showed a positive association with gut barrier function and a negative relation with proinflammatory markers (Fig. 5D).

### C. butyricum-derived extracellular vesicle administration alleviated DSS-induced colitis.

**FIG 6:**
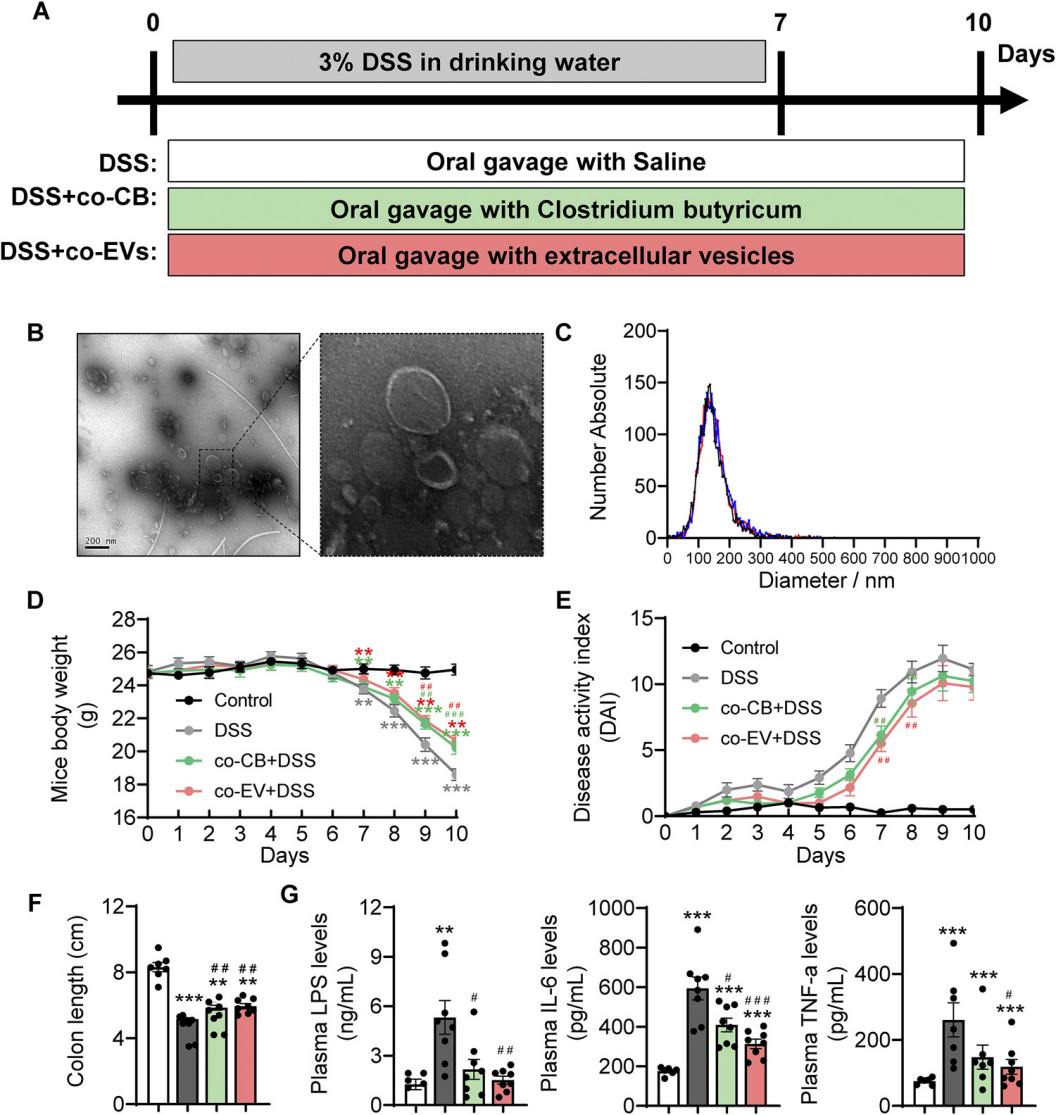
C. butyricum-derived extracellular vesicles administration alleviated colitis. (A) Experimental design. (B) TEM of isolated EVs. (C) Size distribution of EVs analyzed by NTA. (D) Mice body weight (*n *= 8). (E) DAI score. (F) Colon length (*n *= 8). (G) Plasma LPS, IL-6, and TNF-α levels (*n *= 8). *, *P ≤ *0.05; **, *P ≤ *0.01 versus the control group, ^#^, *P ≤ *0.05; ^##^, *P ≤ *0.01 versus the DSS group.

Finally, we investigated how C. butyricum establishes metabolic communication with the host and determines the role of its derived EVs in DSS-induced colitis (Fig. 6A). Transmission electron microscopy (TEM) was used to confirm the presence of EVs in the bacterial cultures (Fig. 6B). EVs were also quantified by NTA (nanoparticle tracking analysis) with a population of 149.3 ± 50.9 nm and possessed a peak range from 27.1 to 445.0 nm (Fig. 6C). The symptoms of mice in each group were measured to explore whether C. butyricum-derived membrane EV administration could alleviate colitis. Compared with the DSS group, mice in the co-CB+DSS and co-EV+DSS groups showed a lower body weight loss, DAI score, and shortened colon length (Fig. 6D to F and Fig. S1D). Moreover, the levels of LPS, IL-6, and TNF-α were significantly reduced in the co-CB+DSS and co-EV+DSS groups (Fig. 6F).

**FIG 7:**
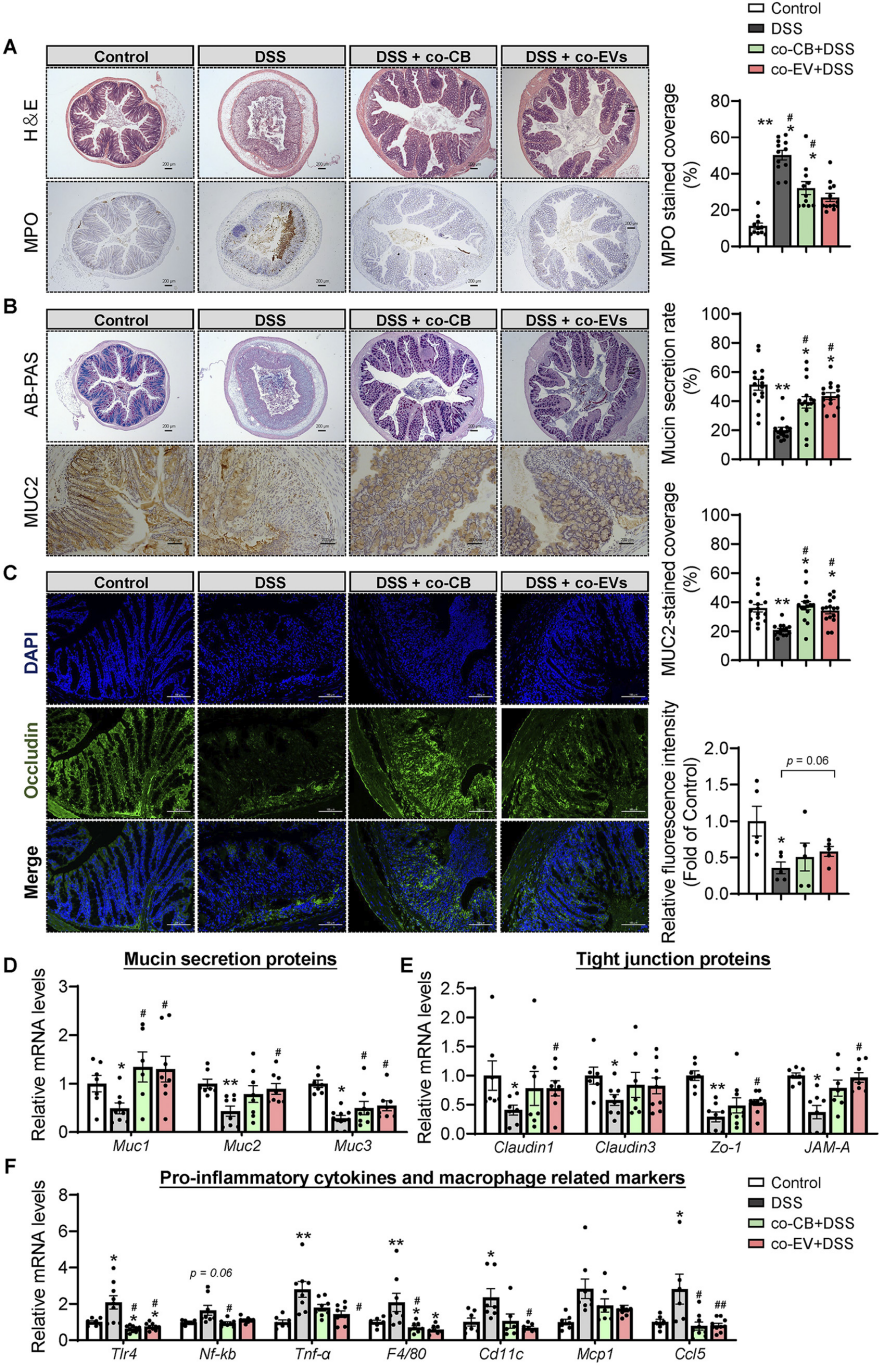
C. butyricum-derived extracellular vesicles administration attenuated the colonic barrier damage. (A) H&E staining of the colon with summarized histological score and immunohistochemical analysis of MPO with summarized MPO stained rate (*n *= 5). (B) Alican blue-PAS staining of the colon with its mucin secretion rate and immunohistochemical analysis of MUC2 with MUC2-stained coverage (*n *= 5). Scale bar: 200 μm. (C) Immunofluorescence staining of tight junction protein Occludin expression in the colonic section from mice (*n *= 5). Blue: DAPI; Green: Occludin. Scale bar: 100 μm. (D) Relative mRNA levels of Mucin secretion proteins, tight junction proteins, and proinflammatory related gene expression (*n *= 7 to 8). *, *P ≤ *0.05; **, *P ≤ *0.01 versus the control group, ^#^, *P ≤ *0.05; ^##^, *P ≤ *0.01 versus the DSS group.

After administration of C. butyricum membrane EVs, we observed reduced inflammatory cell infiltration and mucus layer damage in the colon (Fig. 7A and Fig. S1E). Consistently, cotreatment with CB and EVs largely reversed DSS-induced colonic neutrophil cell infiltration (Fig. 7A). Furthermore, cotreatment with CB and EVs also significantly enhanced mucus barrier integrity, as evidenced by the higher levels of mucin secretion and MUC2-positive coverage (Fig. 7B). Moreover, immunofluorescence analysis of occludin revealed that CB and EV treatment attenuated the DSS-damaged epithelial tight junction protein barrier (Fig. 7C). In line with these findings, DSS decreased the expression of genes involved in mucin secretion (*Muc1*, *Muc2*, *Muc3*, and *Muc4*) and tight junctions (*Claudin1*, *Claudin3*, and *Zo-1*). DSS also increased the levels of *Tlr4* and *Nf-kb* (antibacterial genes) and proinflammatory genes, such as *Tnf-α*, *F4/80*, *Cd11c*, *Mcp1*, and *Ccl5*, while cotreatment with CB and EVs reversed these DSS-induced abnormal changes (Fig. 7D to F). It should be noted that cotreatment with EVs may profoundly alleviate acute colitis symptoms compared to CB treatment.

### C. butyricum-derived extracellular vesicle administration attenuated colonic barrier damage.

**FIG 8:**
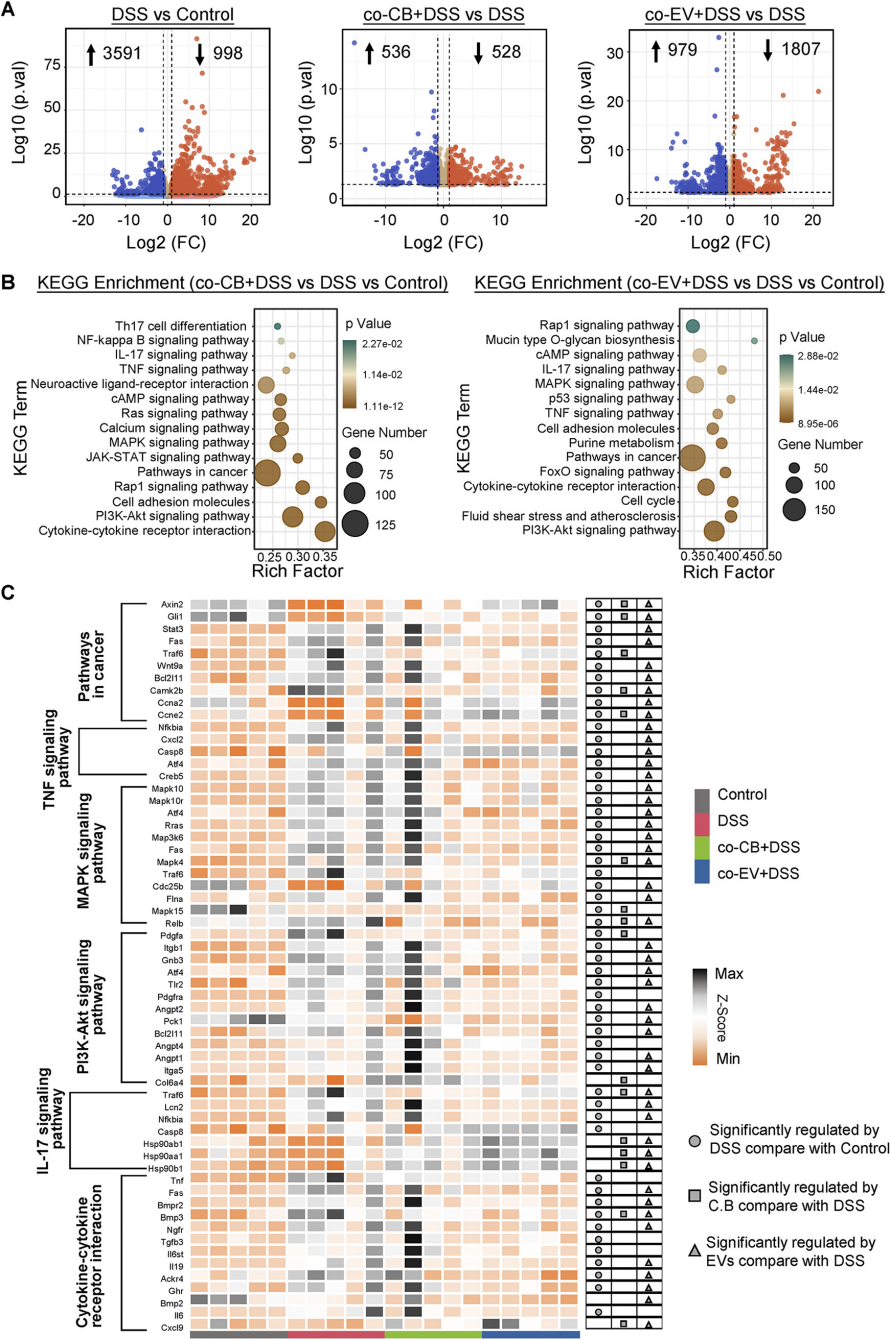
RNA sequencing exhibited distinct colonic function by C. butyricum-derived extracellular vesicles administration. (A) Volcano plot of differentially expressed genes (*n *= 5). (B) KEGG enrichment of pathway. (C) Heatmap of the differentially expressed genes. The scale bar shows the normalized gene expression in each group.

To elucidate the underlying mechanisms of how EVs and C. butyricum reduced the symptoms of colitis, RNA-seq was performed. As shown in Fig. 8, a total of 998 genes were downregulated, while 3591 genes were upregulated in the DSS group compared with the control group. In addition, a total of 528 genes were downregulated and 536 genes were upregulated in the co-CB+DSS group compared with the DSS mice. Moreover, a total of 979 genes were upregulated and 1807 genes were downregulated in the co-EV+DSS group compared with the DSS group (Fig. 8A). KEGG enrichment analysis showed that the most enriched pathways (co-EV+DSS versus DSS versus Control/co-CB+DSS versus DSS versus Control) were both closely related to the inflammatory and immune response, such as the signaling pathways of IL-17, TNF-α, MAPK, PI3K-Akt, and the cytokine-cytokine receptor interaction (Fig. 8B). Furthermore, the NF-κB signaling pathway was the most enriched in the co-CB+DSS versus DSS versus control group, while mucin-type O-glycan biosynthesis, purine metabolism, and the FoxO signaling pathway were enriched in the co-EV+DSS versus DSS versus the control group (Fig. 8B). Ontology (GO) enrichment analysis also revealed that C. butyricum and its EVs were both involved in the regulation of the inflammatory response, positive regulation of the ERK1 and ERK2 cascades, and the immune response, which thus confirmed their functional role in immune and inflammatory modulation in IBD (Fig. S3A). Heatmap analysis further showed that DSS treatment significantly increased the expression of genes in the cancer pathway and immune- and inflammatory-related pathways, while C. butyricum and its EVs ameliorated these increases (Fig. 8C). Interestingly, EVs might exert a more obvious effect in ameliorating DSS-induced colitis.

We also compared the differential regulation between C. butyricum and its EVs. For example, C. butyricum induced 790 downregulated genes and 576 upregulated genes compared with EV treatment (Fig. S3B). Moreover, the differential pathways between these two groups were closely related to amino acid metabolism, especially histidine, tryptophan, arginine, and proline metabolism (Fig. S3C). From the differentially expressed gene analysis, C. butyricum upregulated the expression of genes, including *Camp*, *Adcyap*1, *IL-*17*b*, *Bglap*2, and *Zfp*74, while *Svs*1, *Doxl*2, and *Rik* expression were enhanced by EVs (Fig. S3D).

### C. butyricum-derived extracellular vesicle administration modulated the gut microbiota composition.

**FIG 9:**
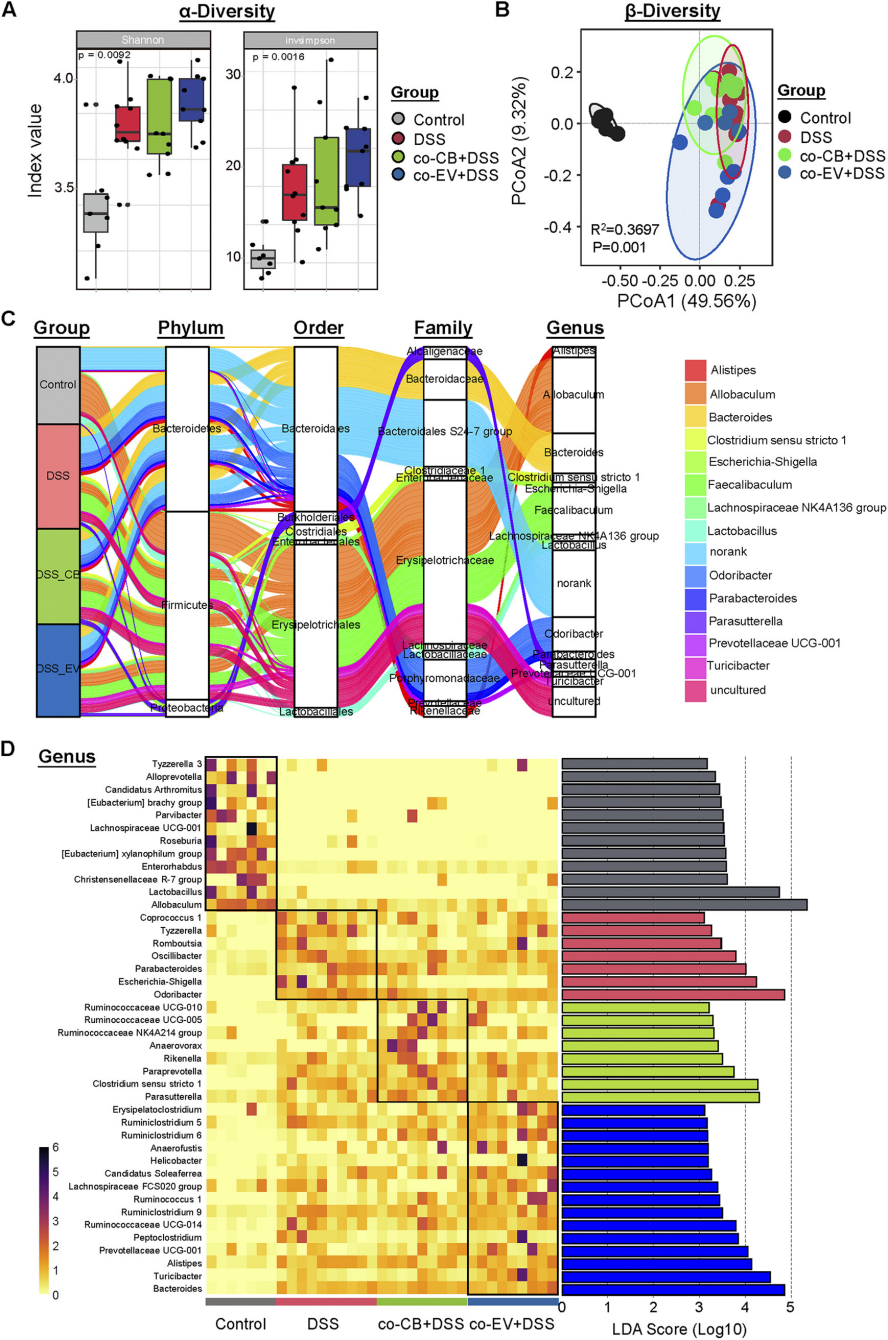
C. butyricum-derived extracellular vesicles administration regulated the gut microbiota composition. (A) α-diversity of Shannon and invsimpson index. (B) PCoA plots among these groups. (C) Sankey plot showed the phylum-genus levels among these groups. (D) Heatmap combined with the analysis of differences in the microbial taxa shown by LEfSe.

The gut microbiota changes upon C. butyricum and its EVs were also analyzed. The Shannon and Invsimpon index showed that the administration of C. butyricum and its extracellular vesicles significantly increased the microbial diversity (Fig. 9A). A separation in the gut microbiota structure was observed between the co-CB + DSS and co-EV+ DSS groups, indicating that the microbiota community in mice with colitis was influenced by C. butyricum and its EVs (Fig. 9B).

*Bacteroidetes*, *Firmicutes*, and *Proteobacteria* were found to be the predominant phyla in the colonic microbiota (Fig. 9C). At the genus level, *Allobaculum*, *Bacteroides,* and *Feacaibaculum* were the dominant bacteria (Fig. 9C). As shown in Fig. 9D, eight genera, *Ruminococcaceae*, *Anaerovorax*, *Rikenella*, and Clostridium sensu stricto-1, were enriched in the co-CB+DSS group, while 15 genera, *Ruminiclostridium*, *Anaerofustis*, *Helicobacter*, and *Alistipes*, were enriched in the co-EV+DSS group by LefSe. Additionally, seven genera, such as Escherichia*/Shigella*, were enriched in the colitis-induced mice (Fig. 9D).

We compared the microbiota community differences between the co-CB+DSS and co-EV+DSS groups. PCoA analysis showed a distinct separation between the two groups (R^2^=0.4081, *P* = 0.007; Fig. S4A). LEfSe further revealed the biomarker bacteria that were enriched in the two groups. For example, *Turicibacter*, *Rodrburia*, and *Romboutsia* were more abundant in the co-EV+DSS group, while short-chain-acid producing bacteria, Clostridium sensu stricto-1, were significantly increased by the C. butyricum treatment (Fig. S3B).

### Correlations among the gut microbiota composition, microbiota function, and colitis-related parameters.

**FIG 10:**
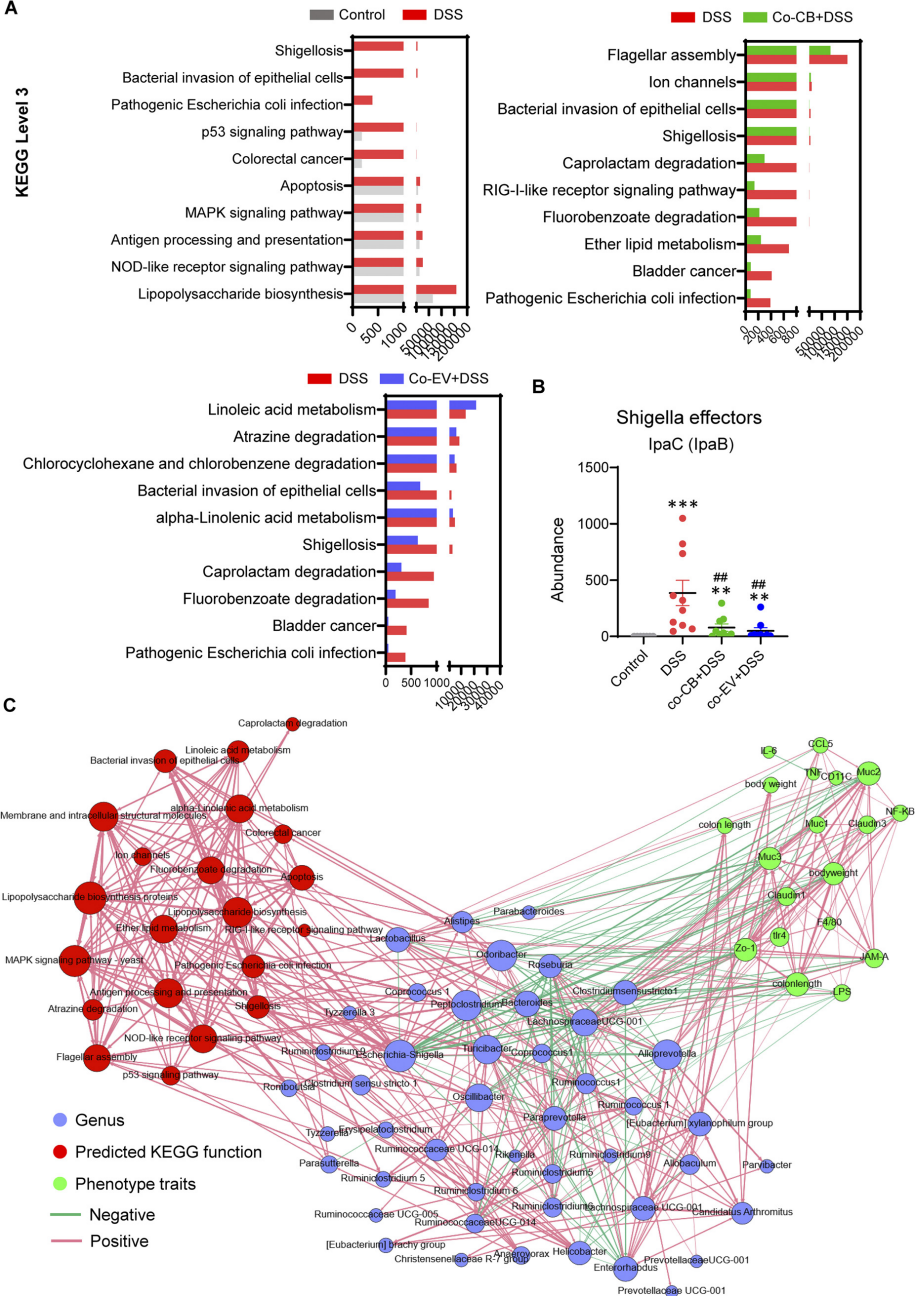
Correlations among changed gut microbiota composition, microbiota function, and altered colitis-related parameters. (A) Microbiota function predicted by PICRUSt2 (control versus DSS group, DSS versus co-CB+DSS group, DSS versus co-EV+DSS group). (B) Shigella effector lpac (lpaB) expression upon each group. (C) Spearman correlation between changed gut microbiota composition, microbiota function, and altered colitis-related parameters (cor > 0.8, *P* > 0.01). *, *P ≤ *0.05; **, *P ≤ *0.01 versus the control group, ^#^, *P ≤ *0.05; ^##^, *P ≤ *0.01 versus the DSS group.

PICRUSt2 was used to predict the microbiota function information derived from metagenomics data (21) (Fig. 10A). Lipopolysaccharide biosynthesis, shigellosis, bacterial invasion of epithelial cells, pathogenic Escherichia coli infection, and other related microbiota functions were enhanced in colitis mice compared with control mice (Fig. 10A). C. butyricum and its EVs significantly reversed these functions, such as shigellosis, bacterial invasion of epithelial cells, and pathogenic E. coli infection (Fig. 10A). Moreover, the Shigella effector abundance was remarkably reduced by C. butyricum and EV treatment, which might contribute to the decreased Escherichia/Shigella abundance at the genus level (Fig. 10B).

Spearman correlation analysis revealed a network within the gut microbiota community, microbiota functions, and altered colitis-related parameters (Fig. 10C). As expected, the relative abundance of Escherichia*/Shigella* was negatively correlated with gut barrier traits and positively correlated with predicted KEGG functions, such as Shigellosis. In addition, Escherichia*/Shigella* was also negatively correlated with *Lactobacillus* (Fig. 10C). Moreover, the abundance of the genus Clostridium sensu stricto-1 increased because of C. butyricum treatment and was demonstrated to be positively associated with mucin protein production but negatively related to LPS levels (Fig. 10C). These results suggested that gut microbiome alterations induced microbiome functional shifts toward reduced pathogenic bacterial biosynthesis and infection, contributing to the alleviation of colitis development.

## DISCUSSION

Butyrate-producing bacteria are characterized by butyric acid production, which has a protective effect against inflammatory disease (22). C. butyricum is reported to be classified in *Clostridium* cluster I and is a group member of butyric acid-producing, Gram-positive, obligate anaerobic bacteria (23, 24). In our study, we determined that the preventative applications of C. butyricum could protect against colitis, as evidenced by a decreased DAI score and histological damage. Furthermore, we confirmed that the gut microbiota and metabolites, which were modulated by C. butyricum, contributed to the attenuation of DSS-induced colitis. This was supported by the effective alleviated effects resulting from the fecal transplant and fecal filtrate from C. butyricum-treated mice. Of note, C. butyricum-derived EVs protected gut barrier function and improved gut microbiota homeostasis, which also reduced ulcerative colitis symptoms.

Reduced pathogenic microorganisms and increased protective bacterial species are normally associated with the development of IBD (25). Multiple studies have been employed to examine the effects of C. butyricum on microbiota shifts. For example, a study revealed that CBM 588 could increase the abundance of *Bifidobacterium*, *Lactococcus* and *Lactobacillus* species to prevent antibiotic-induced dysbiosis (26). In line with this, preadministration of C. butyricum significantly increased the abundance of Clostridium sensu stricto-1, provided energy to the intestinal epithelium, and regulated the immune response (27). Furthermore, the correlation results suggested that Clostridium sensu stricto-1 was positively related to colonic mucin production but negatively associated with proinflammatory cytokine and gene expression. On the other hand, previous research showed that patients with IBD have lower numbers of butyrate-producing *Butyricicoccus* bacteria in their stools (28). As expected, the level of *Butyricicoccus* was increased by the presence of C. butyricum. Escherichia was higher in colitis-induced mice than in healthy ones and thus probably linked to UC incidence (29). Moreover, *Shigella* spp. has been shown to invade and destroy M cells and spread to neighboring enterocytes (30). These results indicated that increased butyrate-producing bacteria and subsequent production of butyrate and other SCFAs, as well as the inhibition of harmful bacteria, might contribute to the attenuation of colitis.

The major gel-forming mucin MUC2 is the predominant structural component of the mucus barrier, which contributes to host protection in the status of intestinal inflammation (31, 32). The association between UC and mucus thickness is well established. For example, UC is accompanied by mucin barrier dysfunction, with a defective mucus layer, depleted goblet cells, and reduced MUC2 sulfate content (33, 34). Under the DSS regimen, we found a thinner, penetrable mucus layer and a rapid reduction in the expression of *Muc2*, while CB administration upregulated *Muc1*, *Muc2*, and *Muc3* expression, further strengthening the mucus layer. In addition, functional abnormalities in tight junctions were also observed in UC and IBD patients (35). Reduced tight junction protein expression, such as *Occludin*, *Claudins*, and *Zo-1*, was found in the DSS group, while CB treatment or FMT of CB-treated microbiota alleviated DSS-induced tight junction barrier dysfunction. These results suggested that the reduced severity of colitis in the CB group may be attributed to the protection of gut barrier integrity.

Xie et al. (36) reported that C. butyricum was able to inhibit TLR2 signaling and IL-17 secretion, further exerting a protective effect on intestinal inflammation induced by DSS. In line with these results, C. butyricum downregulated *Tlr4* and *Nf-κb* expression in colon tissue, accompanied by decreased levels of macrophage markers, such as *F4/80* and *Cd11c*. Moreover, chemokines were reported to be a trigger of multiple inflammatory responses that can mediate intercellular cross-talk to boost the local mucosal immune system (37, 38). Herein, the elevated expression of the chemokines *Mcp1* and *Ccl5* was suppressed by C. butyricum pretreatment, contributing to ameliorated mucosal inflammation and reduced histological damage scores in colitis.

Fecal microbiota transplantation implied the functional role of the gut microbiome (39). A study revealed that sterile filtrates transferred from a donor stool, rather than fecal microbiota, may be sufficient to restore normal intestinal health and eliminate dysbiosis symptoms (40). Thus, to verify the roles of CB-mediated microbiota and metabolites, FMT and FFT from normal mice or CB-treated mice were performed. Consistent with C. butyricum treatment, CB-FMT alleviated the phenotypes of colitis more effectively than FMT in normal mice, indicating that C. butyricum-mediated intestinal microbiota played a pivotal role in colitis alleviation. In addition to FMT, FFT from CB-treated donors alleviated DSS-induced colitis and protected against proinflammatory gene expression and gut barrier dysfunction. This was in accordance with a recent study involving human patients successfully treated with FFT to cure a Clostridium difficile infection (40). The reduced *Akkermansia* abundance in IBD patients has been associated with impaired intestinal barrier function (41). Similarly, *Akkermansia* was enriched in the FMT of CB-treated microbiota but absent in the CB-FFT group. Moreover, the correlation analysis suggested that *Akkermansia* was positively linked to mucin secretion but negatively associated with proinflammatory gene expression. On the other hand, *Lactobacillus*, one of the most common probiotics, was found to be significantly increased in the CB-FFT group and had a clear negative correlation with the inflammatory index. However, these results showed that CB-FMT and CB-FFT were more similar to the control group than to the C. butyricum-only treatment. Notably, the function of the altered gut microbiota and its derived metabolites could not be ignored.

EVs released by the microbiota have a profound impact on the host by transporting and delivering effector molecules that may modulate host signaling pathways and varied cell processes (42). To explore the crosstalk between C. butyricum and the host, we assumed that EVs derived from C. butyricum may also be able to mediate interactions with the host. Herein, we focused on the role of EVs from C. butyricum in protecting against colitis. As expected, C. butyricum and its derived EVs ameliorated colitis symptoms, improved histological scores, and enhanced intestinal barrier function and inflammatory responses. Specifically, EVs provided a beneficial effect by reinforcing the epithelial barrier through regulation of the TJ proteins *Claudins*, *Zo-1,* and *JAM-A*. In line with a previous study, treatment with EVs also reduced the expression of proinflammatory genes, such as *Tlr4*, *Tnf-α*, *Cd11c,* and *Ccl5* (43). It was reported that bacteria-derived EVs involved in host-microbe responses modulate the gut microbiota (44). Oral administration of EVs increased the presence of the beneficial bacteria *Ruminiclostridium* and *Ruminococcaceae* UCG-014 in the gut. These are known to secrete short-chain fatty acids and help maintain the functionality and morphology of the intestinal epithelial barrier (45). Collectively, these findings highlight the crucial role of functional probiotic-derived EVs and their influence on immune response amelioration and intestinal homeostasis regulation.

Because EVs seem to contribute more to alleviating colitis than C. butyricum alone, we decided to investigate the difference between C. butyricum and EVs. LEfSe showed that the genus *Roseburia*, the dominant butyrate-producing bacteria that exert anti-inflammatory properties, was more enriched in the EV treatment group than in the co-CB+DSS group (46). However, Clostridium sensu stricto-1 was dominant in the co-CB+DSS group, which was in line with the Pre-CB group. Notably, RNA-seq revealed that EV treatment showed a tendency to enhance amino acid production, especially tryptophan. Because tryptophan was found to have an important role in the regulation of immunity, neuronal function and intestinal homeostasis, treatment with EVs would presumably benefit such functions, resulting in the alleviation of colitis symptoms. On the other hand, the enhanced actions of EVs might be due to their ability to diffuse into the colonic tissue and other distal tissues, where they would directly exert regulatory effects, while the effects of C. butyricum would be more localized. This investigation shows promise toward developing a possible treatment for colitis. Thus, continuing this research to understand more about such mechanisms would be most valuable.

In conclusion, these findings demonstrated that C. butyricum supplementation is a potentially effective strategy for IBD therapy. The beneficial effects of C. butyricum were attributable to its close association with mediating the gut microflora to a normal microbial community and metabolite content. Additionally, C. butyricum-derived EVs helped to modulate intestinal homeostasis, resulting in the alleviation of colitis symptoms. These effects may result from a reduction of Escherichia/*Shigella* levels, enhancing gut barrier integrity and suppressing the inflammatory response. Finally, our study provides new insights into C. butyricum-mediated IBD prevention and promotes the development of novel therapeutic and preventive interventions for IBD treatment.

## MATERIALS AND METHODS

### EVs isolation and identification.

Clostridium butyricum MIYAIRI II 588 was obtained from Miyarisan Pharmaceutical Co, Ltd. (Tokyo, Japan). C. butyricum was cultured under anaerobic conditions at the temperature of 37°C. The EVs were isolated from the bacterial culture supernatants as previously described (47). In brief, bacterial cultures were centrifuged at 8,000 g for 30 min, the supernatant was collected and centrifuged at 20,000 g for 45 min and subsequently filtered aseptically through a sterile 0.22-μm bottle-top filter. The filtrate was centrifuged by ultracentrifugation in a 45 Ti rotor (Beckman Coulter, Fullerton, CA, USA) at 120,000 g for 2 h at 4°C. Then, the pellet was resuspended in phosphate-buffered saline (PBS) and centrifuged again at 120,000 g for 2 h. The EVs were finally resuspended in PBS and collected for further study. Isolated EVs were visualized by transmission electron microscopy (TEM). The size distribution was analyzed by ZetaView PMX 110.

### The first animal trial: the preventive effect of C. butyricum on colitis.

Male C57BL6J mice were kept in the temperature-controlled room (21 ± 3°C) under a 12h-12h dark-light cycle. After 1 week of acclimation, the mice were allocated into groups according to their body weight. Colitis was induced by the administration of DSS (MW: 36 − 50 kDa, Yeasen Biotech Co., Ltd., Shanghai, China) through the drinking water. To investigate the preventive impact of C. butyricum on colitis, the mice were pretreated with or without C. butyricum (10^8^ CFU) for 21 consecutive days and then given 3% DSS via drinking water for another 7 days followed by 3 days of recovery, *n *= 7 to 8 for each group. All animal procedures were approved by the Institutional Animal Care and Use Committee of the Zhejiang Academy of Agricultural Sciences (2018ZAASLA20).

### The second animal trial: the involvement of C. butyricum-mediated microbiota and metabolites on colitis.

To further determine the involvement of microbiota and metabolites in C. butyricum mediated in the attenuation of colitis, the FMT and FFT were conducted as described previously (48, 49). For FMT and FFT, fresh feces from each group were pooled and homogenized, diluted in sterile saline with a final concentration of 50 mg feces/mL. Pooled samples were centrifuged at 100 × *g* for 2 min, collect the supernatant, and then centrifuged at 300 × *g* for 5 min. The supernatant was filtered through a 70 μm filter and was used for FMT treatment. For FFT, the supernatants were collected and passed through 70 and 0.22 μm filters. FMT or FFT was administered per mouse via oral gavage (200 μL) every day during the DSS experiment for 10 days.

### The third animal trial: the effect of C. butyricum-derived extracellular vesicles on colitis.

To assess the effect of C. butyricum-derived EVs on colitis, the mice were divided into the following four groups: (i) Control group: mice were gavaged with 200 μL and normal drinking water, *n *= 7; (ii) DSS group: mice were given 3% DSS drinking water and orally gavaged with saline, *n *= 8; (iii) co-CB+DSS group: mice were administered 3% DSS and orally gavaged with C. butyricum (10^8^ CFU), *n *= 8; (iv) co-CB+DSS group: mice were administered 3% DSS and gavaged with the EVs (50 μg/day), *n *= 8. Fresh DSS solution was provided every 2 days until the end of the study. Body weight was measured daily during the whole duration of the study. The mice were sacrificed after anesthesia on day 11. The colon of each mouse was excised and measured.

### Disease activity index (DAI) and histopathological assessment.

DAI was evaluated to assess the severity of the colitis according to methods described previously (50). For morphological measurements, formalin-fixed colon tissues were stained with Alcian blue/periodic acid-Schiff (AB/PAS) and hematoxylin and eosin (H&E). Histopathological assessment was calculated as described previously (50).

### Measurement of inflammatory factors in serum.

The inflammatory state of serum was assessed through the measurement of mouse LPS (CSB-E13066m, CUSABIO, https://www.cusabio.com/) and the levels of inflammatory cytokine assay such as mouse IL-6 (EK206/3, MultiSciences) and TNF-α (EK282/4, MultiSciences) according to the manufacturer’s recommendations.

### Real-time quantitative PCR.

The total RNA from the colon tissues was extracted by using TRIzol (azyme Biotech Co., Ltd.). Total RNA was purified by lithium chloride precipitation (51). RT-PCR was performed with 2×ChamQ SYBR Color qPCR Master Mix (Vazyme Biotech Co., Ltd.) according to the manufacturer’s instructions. Relative mRNA expressions were quantified using the threshold cycle (2^−ΔΔCT^) method as described previously (49). The primers are shown in Table S1.

### Immunohistochemistry (IHC) and immunofluorescence staining (IF).

IHC and IF analyses were performed as described previously (49). Primary antibodies for IHC and IF were as followed: anti-MUC2 (GB14110; 1:500; Servicebio); anti-MPO (GB12224; 1:200; Servicebio); anti-Occludin (GB111401; 1:200; Servicebio). Data were analyzed by Image J.

### 16s rRNA gene sequencing.

QIAamp DNA isolation kit (Qiagen, Hilden, Germany) was used to extract the total genomic DNA of colonic content based on the manufacturer’s instructions. The highly variable V4 to V5 region of the 16S rRNA gene was amplified. Raw data were analyzed by the QIIME2 platform. The metagenomes prediction was made with PICRUSt2. The gene content was predicted for each sample according to KEGG. More detailed bioinformatics methods can be found in a previous study (52).

### Colonic transcriptional profiles for Trail 3.

TRIzol reagent was used to extract RNA from the colonic sample following the manufacturer's instructions. The RNA libraries were sequenced on the Illumina Novaseq™ 6000 platform by LC Bio Technology CO., Ltd. (Hangzhou, China). Briefly, the total RNA quantity and purity were analyzed using a Bioanalyzer 2100 and RNA 6000 Nano LabChip kit (Agilent, CA, USA, 5067-1511), and high-quality RNA samples with RIN number >7.0 were used to construct a sequencing library. Genes differential expression analysis was performed by DESeq2 software. Pathway enrichment analysis identified significantly enriched metabolic pathways or signal transduction pathways in DEGs compared with the whole-genome background.

### Statistical analysis.

Data were represented as means ± SEM. Student's *t* test and one-way ANOVA were performed to analyze the differences by using the Prism 9.0 program (GraphPad Software, San Diego, Canada). The adjusted *P*<0.05 indicates statistically significant. Spearman correlations were carried out using the R statistical software (R version 3.5.3).

### Data availability.

The 16S rRNA gene and RNA sequencing data were submitted to the NCBI SRA database under the study accession numbers, PRJNA795271 and PRJNA795830.
