# Supplementary material for: Clostridium butyricum and Its Derived Extracellular Vesicles Modulate Gut Homeostasis and Ameliorate Acute Experimental Colitis
Source: Microbiol Spectr. 2022 Jun 28;10(4):e01368-22. doi: 10.1128/spectrum.01368-22 (PMC9431305; doi:10.1128/spectrum.01368-22)
Supplement: Supplemental file 1 — Supplemental material. Download spectrum.01368-22-s0001.pdf, PDF file, 0.6 MB [file spectrum.01368-22-s0001.pdf]

Supplementary Figure 1

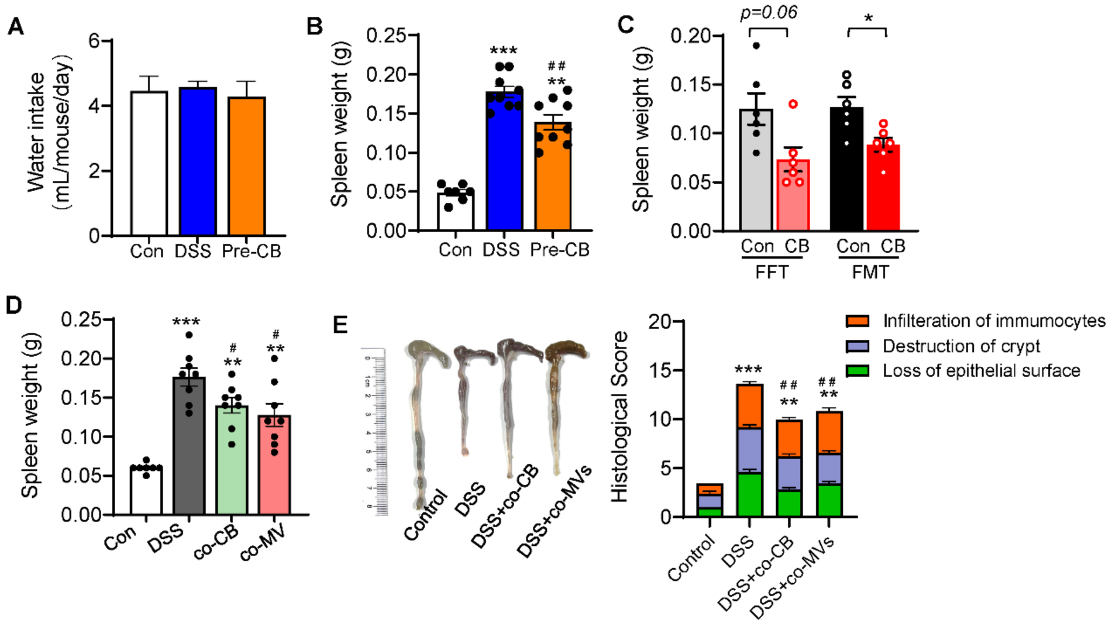

Supplementary Figure 1. (A) Water intake. (B) Spleen weight for *C. butyricum* pre-treatment. (C) Spleen weight for FMT and FFT. (D) Spleen weight for the part 3 experimental. (E) Representative picture for colon length in the part 3 experimental and histological score for part 3 experimental. Data are represented as mean ± SEM, (* $p \leq 0.05$, ** $p \leq 0.01$ vs the control group, $^{\#}p \leq 0.05$, $^{\#\#}p \leq 0.01$ vs the DSS group).

Supplementary Figure 2

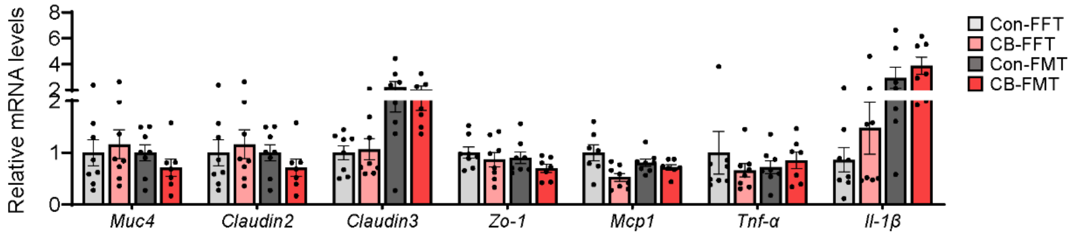

Supplementary Figure 2. Relative mRNA levels genes in the colon tissues of FFT and FMT experimental.

Supplementary Figure 3

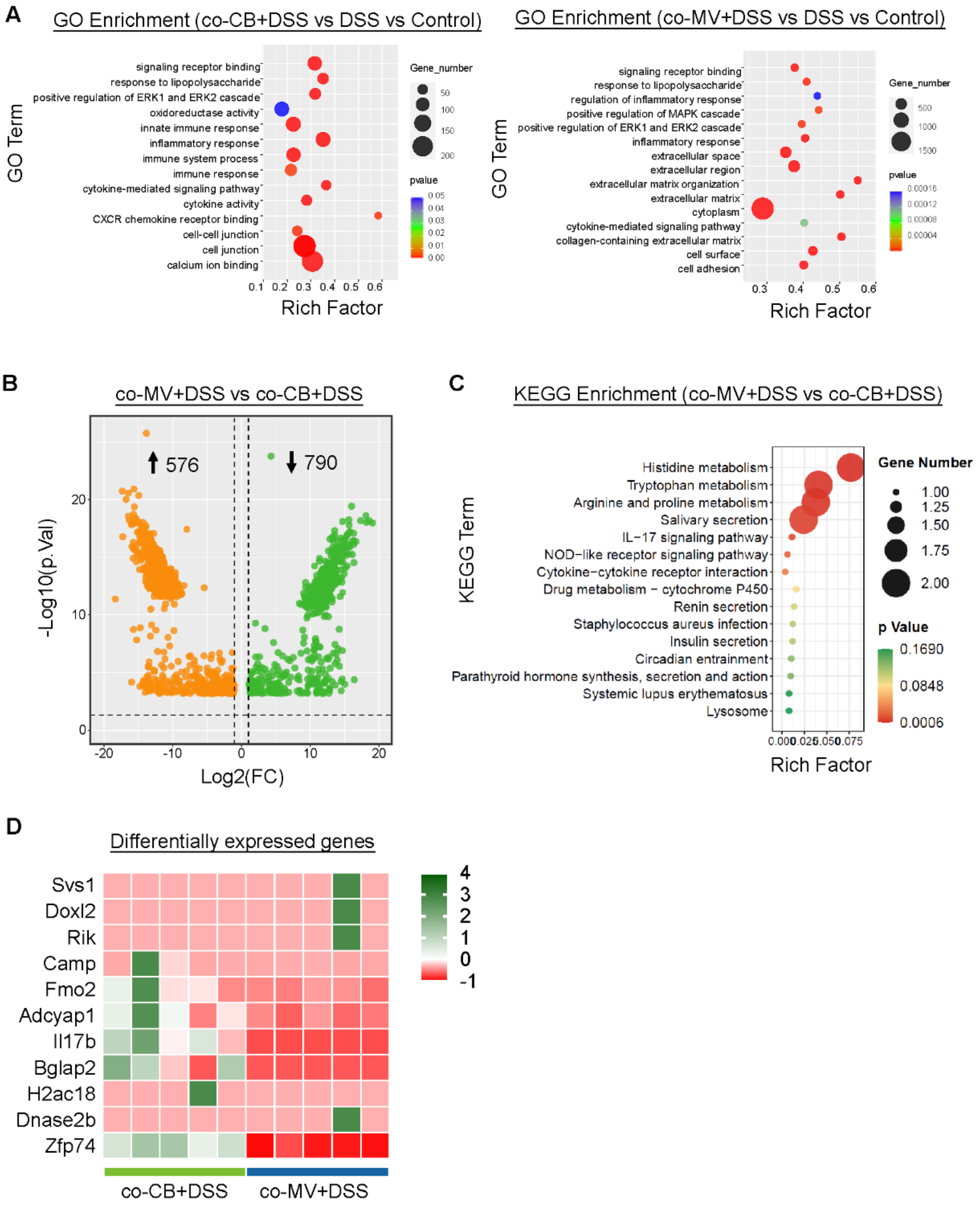

Supplementary Figure 3. (A) GO enrichment of pathway in co-CB+DSS vs DSS vs Control group, co-MV+DSS vs DSS vs Control group. (B) Volcano plot of differentially expressed transcripts with co-MV+DSS vs co-CB+DSS group. (C) KEGG enrichment of pathway in co-MV+DSS vs co-CB+DSS group. (D) Heatmap summary of the differentially expressed genes.

Supplementary Figure 4

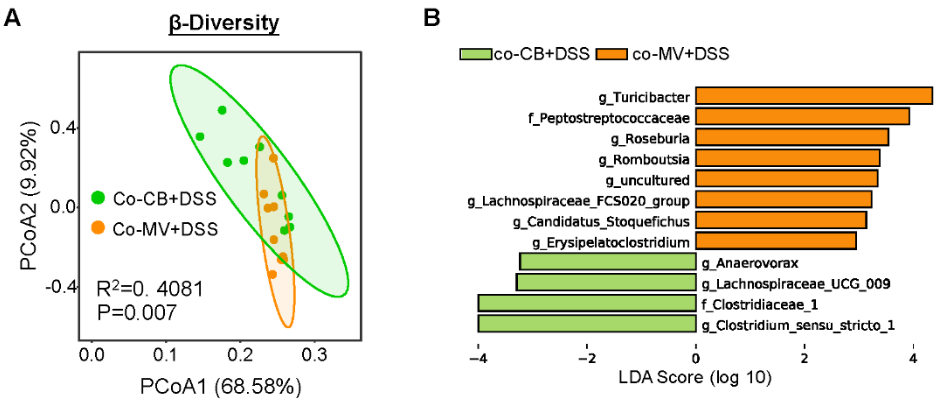

Supplementary Figure 4. (A) PCoA of gut microbiota in each group. (B) Analysis of differences in the microbial taxa shown by LEfSe.

Supplementary Table S1. Mice primer sequences in RT- PCR assays.

**Genes**	**Forward**	**Reverse**
mβ-Actin	AGGCCCAGAGCAAGAGAGGTA	GGGGTGTTGAAGGTCTCAAACA
mF4/80	GCTGTGAGATTGTGGAAGCA	CTGTACCCACATGGCTGATG
mCd11c	AAAATCTCCAACCCATGCTG	CACCACCAGGGTCTTCAAGT
mTnf-a	CCCACACCGTCAGCCGATTT	GTCTAAGTACTTGGGCAGATTGACC
mlL-6	CCACTTCACAAGTCGGAGGCTTA	GCAAGTGCATCATCGTTGTTCATAC
mlL-1β	GGGCCTCAAAGGAAAGAATC	TACCAGTTGGGGAACTCTGC
mMcp1	AGGTCCCTGTCATGCTTCTGG	CTGCTGCTGGTGATCCTCTTG
mCcl5	TGCCCTCACCATCATCCTCACT	GGCGGTTCCTTCGAGTGACA
mClaudin 1	GTTTGCAGAGACCCCATCAC	AGAAGCCAGGATGAAACCCA
mClaudin 2	GTC ATC GCC CAT CAG AAG AT	ACT GTT GGA CAG GGA ACC AG
mClaudin 3	GCT CTC AGA GTC CGT TGA CC	CTG CCC TTT CAG GTT AGC AG
mJAM-A	CACCTTCTCATCCAGTGGCATC	CTCCACAGCATCCATGT-GTGC
mZo-1	AAGAATATGGTCTTCGATTGGC	ATTTTCTGTCACAGTACCATTTATCTTC
mMuc1	AGTTACGGTCAGGCTGCTCCGTGGT	ACCCTCCCGGAAAACCACAGTC
mMuc2	TGCTGCTGACGAGTGGTTGGTG	CGGACGCTTGGTGGTGAGGC
mMuc4	GGCCCGCTTGGACATTTGGTGA	AGTCTCCCAGCCCGTTGAAGGT
